# Undergraduate medical textbooks do not provide adequate information on intravenous fluid therapy: a systematic survey and suggestions for improvement

**DOI:** 10.1186/1472-6920-14-35

**Published:** 2014-02-20

**Authors:** Arfon GMT Powell, Simon Paterson-Brown, Gordon B Drummond

**Affiliations:** 1 Unit of Experimental Therapeutics, College of Medical, Veterinary and Life of Sciences, University of Glasgow, 2nd Floor McGregor Building, Western Infirmary, Glasgow, G11 6NT, Scotland; 2 Department of Surgery, Royal Infirmary of Edinburgh, 51, Little France Crescent, Edinburgh, EH16 4SA, Scotland; 3 Department of Anaesthesia and Pain Medicine, The University of Edinburgh, Royal Infirmary of Edinburgh, 51, Little France Crescent, Edinburgh, EH16 4SA, Scotland

**Keywords:** Fluid Therapy, Textbooks, Teaching, Medical graduates

## Abstract

**Background:**

Inappropriate prescribing of intravenous (IV) fluid, particularly 0.9% sodium chloride, causes post-operative complications. Fluid prescription is often left to junior medical staff and is frequently poorly managed. One reason for poor intravenous fluid prescribing practices could be inadequate coverage of this topic in the textbooks that are used.

**Methods:**

We formulated a comprehensive set of topics, related to important common clinical situations involving IV fluid therapy, (routine fluid replacement, fluid loss, fluids overload) to assess the adequacy of textbooks in common use. We assessed 29 medical textbooks widely available to students in the UK, scoring the presence of information provided by each book on each of the topics. The scores indicated how fully the topics were considered: not at all, partly, and adequately. No attempt was made to judge the quality of the information, because there is no consensus on these topics.

**Results:**

The maximum score that a book could achieve was 52. Three of the topics we chose were not considered by any of the books. Discounting these topics as “too esoteric”, the maximum possible score became 46. One textbook gained a score of 45, but the general score was poor (median 11, quartiles 4, 21). In particular, coverage of routine postoperative management was inadequate.

**Conclusions:**

Textbooks for undergraduates cover the topic of intravenous therapy badly, which may partly explain the poor knowledge and performance of junior doctors in this important field. Systematic revision of current textbooks might improve knowledge and practice by junior doctors. Careful definition of the remit and content of textbooks should be applied more widely to ensure quality and “fitness for purpose”, and avoid omission of vital knowledge.

## Background

Some practical aspects of medical education in the UK appear to be deficient. For example, a questionnaire study of 710 consultant surgeons in the UK, on intravenous fluid prescribing showed that only 16% felt that trainee doctors had been adequately trained in prescribing intravenous fluids, and only 30% of them judged that patients after surgery received appropriate water and electrolyte therapy [1]. Intravenous fluid therapy is fundamental in medical care, and poor management increases complications [2-5]. Fluid overload can cause peripheral oedema, reduce the strength of surgical anastomoses [6], and even lead to pulmonary oedema and death [7-11]. Recent UK National Clinical Guideline Centre guidelines on intravenous therapy suggest that over-hydration occurs in 17-54% of patients and this causes complications in up to 50%. The guidelines conclude that “Lack of adequate clinician preparation is associated with potential for increased clinical risk and harm” [12]. Prescription of such therapy is often left to junior members of the surgical team, who therefore should have sufficient knowledge of this important topic.

“Tomorrow’s Doctors”, published by the General Medical Council [13], sets a broad syllabus for medical training in the UK, and is regularly updated. Although this explicitly specifies teaching topics such as the recognition and management of acute illness, the provision of proper surgical and peri-operative care, and oxygen therapy, the topic of intravenous fluids is not mentioned. Why are junior doctors poorly informed on this topic? Textbooks primarily aimed at anaesthetic trainees cover this topic badly [14]. Because this topic should be covered adequately in basic medical teaching, we systematically reviewed information provided regarding IV fluid therapy in commonly used undergraduate textbooks. Our aim was to discover if textbook information was deficient, which could possibly explain the poor knowledge noted by clinicians. These topics are shown in the list of questions section. We found that many textbooks did not adequately address these basic features of intravenous therapy.

### Adequacy of textbook coverage: does the book provide information about the following topics

#### Description of fluid and electrolyte homeostasis

Fluid and electrolyte requirements for a standard adult

Fluid balance, including losses and gains in a healthy adult

Electrolyte composition of different body fluids

Are the routes of perioperative fluid loss described

#### How does surgery contribute to fluid loss

Systemic Inflammatory Response

Increased ADH secretion

Type of surgery and specific effects on fluid balance.

Evaporation during a laparotomy

Normal physiological changes seen in a post-operative patient

#### Patient assessment and assessment of losses

How is blood volume assessed in the routine postoperative patient

Signs and symptoms of blood loss in the patient after surgery

How are perioperative losses assessed

Signs of fluid overload in the patient after surgery

Signs of inadequate circulating volume in a patient after surgery

Does the book describe how to assess the adequacy of therapy

#### Treatment of normal/losses/excess

Does the book describe intraoperative fluid management

Suitable fluid regiment for 1 day or three days fasting

Reason and evidence for a particular choice of fluids

Fluid resuscitation in a patient with GI losses

Fluid resuscitation in a patient with blood loss

Fluid resuscitation in a patient who is septic

#### Electrolyte content of fluid preparations

Contents of:

5% Glucose

Saline

Hartmanns/Sodium Lactate

Gelofusine (different formulations)

Human albumin solution

## Methods

Before we started our survey, we used an iterative checklist to draw up a formal set of topics that we considered would give sufficient information to allow an adequate clinical assessment and an appropriate IV fluid prescription in a variety of common clinical settings (Table 1). These included the topics of routine perioperative care, fluid overload, hypovolaemia and sepsis.

**Table 1:** The textbooks surveyed: They are not necessarily the most recent editions, but all were published in or after 2002

**Title**	**Author/editor**	**Edn.**	**Pages**
Clinical surgery	eds Henry MM Thompson JN	2	806
Oxford handbook for the Foundation Programme	Hurley N	2	695
Principles and practice of surgery	eds Garden OJ Bradbury AW Forsythe J	4	633
Oxford handbook of clinical medicine	eds Longmore M Wilkinson I Rajagopalan S	6	874
Oxford handbook of clinical specialties	Collier J Longmore M Turmezei T Mafi A	6	807
Acute clinical medicine	eds Kumar P and Clark M	2	742
Surgery at a glance	Grace PA Borley NR	3	189
Oxford handbook of acute medicine	Ramrakha PS	3	869
Care of the critically ill surgical patient	ed Anderson ID	2	159
Crash course: General medicine	Parker R Sharma A	3	531
Renal and urinary system and electrolyte balance	Stamoulos P Bakalis S		120
Crash course: Surgery	Sweetland H Conway K	2	322
ABC of resuscitation	eds Colquhoun MC, Handley AJ Evans TR	5	111
Lecture notes on general surgery	Ellis H Calne RY Watson CJE	10	392
Lecture notes on clinical medicine	Rubenstein D Wayne D Bradley J		360
Key topics in critical care	Craft T Nolan J Parr M	2	268
Lecture notes. Emergency medicine	Moulton C Yates D	3	441
Medicine at a glance	Davey P	3	490
Fluids and electrolytes demystified	Johnson JY		227
Davidson's principles and practice of medicine	eds Boon NA Colledge NR Walker BR		1381
Crash course: Renal and urinary systems	Thomas R Stanley B Datta S	3	210
Oxford handbook of critical care	Singer M Webb AR	2	605
Acute and critical care medicine at a glance	Leach R	2	143
The renal system at a glance	O'Callaghan CA	3	127
Crash course: Metabolism and nutrition	Lim MY Roach JO	3	278
The gastrointestinal system at a glance	Keshav S		117
Oxford textbook of medicine	Warrell DA Cox TM Firth JD Benz EJjr	4	>5000
Emergency medicine. The principles of practice	ed Fulde GWO	4	642
Fundamentals of anaesthesia	eds Smith T Pinnock C Lin T		963

We did not seek ethical approval for the study. With the help of our medical library staff, we compiled lists of undergraduate medical textbooks on surgery, general medicine and critical care, from university libraries and medical publishers in the UK. From these lists we selected 29 textbooks published between 2002 and 2010. We chose all the books that were likely to be used for guidance on fluid balance and IV fluid prescription by medical students in the final years of their course (Table 1).

### Process

An academic surgical trainee with a surgical membership (AGMTP) then surveyed each book separately to find any information on each topic in this working definition of necessary content. The book was searched using chapter headings and index, using the following phrases, separately and in combination: fluid management; perioperative fluid; intraoperative fluid; postoperative fluid; fluid balance; fluid maintenance; maintenance fluids; fluid therapy, intravenous fluids, dehydration, hypovolaemia, losses, abnormal losses, haemorrhage, oedema, fluid overload, sodium chloride, Hartmann’s solution, Sodium lactate, lactated Ringer, balanced salt solution, albumin, colloid, and Gelofusine.

### Scoring

The amount of information that could be found was categorised either as not found, adequately covered, or good coverage. No attempt was made to judge the *quality* of the information, because this was considered to be too subjective to be precisely scored: the score related solely to the *extent* of the coverage. To assess the reliability of this review, an experienced academic anaesthetist (GBD) independently re-scored 20% of textbooks. For the duplicate assessments by the two book assessors, the Spearman coefficient of Rank concordance was 1.0 (P = 0.0167).

Because larger books can cover topics more fully, we also considered the number of pages in each book (taken in all but one case from the description in the British Library Catalogue ( http://explore.bl.uk/primo_library/libweb/action/search.do).

## Results

### Comparison of textbooks

The performance of the textbooks on each topic is illustrated in Table 2. If scores are allocated to each book, using a score of 1 for adequate and 2 for good coverage of each topic, then a book that covered all the topics fully would gain a total score of 52. However, three topics were not covered by any of the books. These were in the section on “How surgery contributes to fluid loss”, where no book considered either the influences of the type of surgery on fluid loss, or evaporation from the surgical field. The third topic not covered by any book was the question on intraoperative fluid management.

**Table 2:** Adequacy of coverage of each topic specified in the checklist

**Normal physiology**	**Average mark**
Standard requirements	0.72
Fluid balance	0.86
Body fluids	0.31
Routes of loss	0.24
**Effects of surgery**	
Systemic inflammation	0.34
ADH	0.41
Types of surgery	0
Evaporation	0
Normal postop changes	0.38
**Assessment of losses**	
Assess blood volume	0.86
Signs of blood loss	0.59
Perioperative loss	0.31
Overload	0.72
Hypovolaemia	0.90
Adequacy of therapy	0.66
**Treatment**	
Intraoperative	0
Suitable regimes	0.31
Reasons for therapy	0.41
Resuscitate GI losses	0.66
Resuscitate Blood loss	0.90
Resuscitate sepsis	0.83
**Fluid preparations**	
5% Glucose	0.55
0.9% w/v saline	0.66
Hartmanns/Sodium lactate	0.59
Gelofusine	0.34
Human albumin solution	0.28

These are averages of scores given for the different topics, using the following score allocation: 0 for no mention, 1 for adequate coverage, 2 for good coverage of the topic.

**Figure 1:**
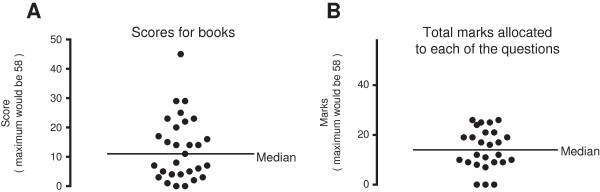
**Panel A: Results of the survey: distribution of total scores gained by the different books. Panel B**: The total marks allocated to the different questions. Three questions were not addressed by any of these books and therefore scored no marks at all.

These topics may not be entirely suitable for an undergraduate book, nor are they directly relevant to management after surgery. If we make a specific discount of these topics, then the maximum score is reduced to 46. In relation to this revised maximum, one book scored very well with a total score of 45. However, the overall median score for all the books was only 11, and the upper and lower quartile values were 4 and 21. (Figure 1A) Out of the 29 books assessed, 14 scored very badly with less than 10 marks (22%).

### Coverage of different topics

Considering the topics that we identified as relevant to routine management, the degree with which each topic was covered varied markedly. As stated above, three topics were not covered by any of the books. Some were covered at least partially by 20 of the 29 books. If all the books (29 books) had provided information on a specific topic sufficiently, then the number of marks attributed to that topic could be 58. The range of marks allocated to each topic ranged from 0 to 21, with a median of 14 (quartiles 9, 21), equivalent to 24, (15–36)%. These medians and ranges, excluding the three “unanswered” topics, indicate that the other topics had a symmetrical range of severity. The average score (14.3) of all the topics was poor, and few of the topics were answered adequately by all the books (Figure 1B). However, since one textbook considered almost all the topics well, the topics cannot be considered too “difficult” or “out of the way”.

The topics on the identification of hypovolaemia and the resuscitation of a patient with blood loss were well covered, with 26 marks allocated out of a maximum of 58 marks possible if all the books had provided a full coverage of the topic. In contrast, the topics that were badly covered were body fluid composition, routes of loss, perioperative loss, and suitable regimens for a patient not taking oral intake for 1 or 3 days. There was a weak relationship between the number of pages and the mark that the book was given (Spearman’s r = 0.43, *P* = 0.022). Books with less than 350 pages generally got very poor marks (average 8.7, or 19%), although two, with less than 200 pages, came second and fifth best, with marks of 29 and 23 (63 and 50%). The marks given to each book for each question are shown in Additional file 1.

## Discussion

Using a simple pre-determined scoring system to assess coverage of topics important for basic management of intravenous fluid therapy we found most undergraduate textbooks were inadequate, with an average score of about 24% of the total possible. However, some books, not necessarily the largest, scored well.

Three of the topics that we defined were not answered by any of the books. These topics were included because a recent exhaustive review [15] suggested that these specific considerations require revision, particularly in view of recent changes in surgical practice such as reduced fasting times, enclosing exposed viscera, and changes in surgical methods to reduce tissue damage.

Knowledge of intravenous fluid use found in trainee doctors at the start of their postgraduate training is poor, and this appears to be a national problem [16]. Clearly, undergraduate teaching fails this subject. One reason for poor education may be the poor coverage of the subject by most textbooks. It is not clear why these textbooks should be so poor, although the generally low marks scored by shorter books show that “quick fix” books are inadequate. Unfortunately these are often the books of recourse for the stressed, who hope that they will “give the facts” more easily and quickly.

Perhaps some books fail to consider some topics because the facts that were sought were considered too elementary to mention, or because some aspects were overlooked by the authors because they are considered “second nature” to an experienced clinician. This is speculation, but we can offer no alternative explanation. It is disturbing that although the “straightforward” and perhaps more “interesting and exciting” topics of blood loss and replacement were tackled successfully, the more mundane topics, which are those that often cause problems, such as postoperative fluid loss and suitable replacement regimens, were so comprehensively neglected. Possibly, well-established books have been taken as a template to provide the subject matter for others written subsequently, so that important relevant topics become overlooked. We could find no evidence that books which had been revised frequently (i.e. had been through several editions) were any better or worse than newly published books. In addition, recognising the *absence* of a topic can be demanding if an agenda or curriculum is not specifically constructed beforehand. Ideally, those who review manuscripts, at all stages, should construct a defined “ideal content list” before they start reading. If this is not done, attention inevitably falls on what is written, and deficiencies are not detected, because reviewers may only consider topics that are explicit. A good review should start with a working definition of necessary content. Similar deficiencies have been found in textbook coverage for other practical issues such as assessment of critically ill patients [17]. If core knowledge of this sort is missing, those who teach and set examinations are faced with difficulty. Textbooks cannot necessarily be used to indicate necessary knowledge, and arguing that material that is not in the textbooks should not be subject to examination is clearly wrong. Currently, our faculty place students approaching graduation to “shadow” junior doctors. This may be an effective teaching process, but only if the junior doctors are themselves sufficiently knowledgeable. The evidence, which we cite above, does not support this assumption, and when clinical practice is inadequate, teaching and assessment may require reappraisal.

In 2008, consensus guidelines were developed to help doctors prescribe IV fluids in a wide range of clinical settings. The British Consensus Guidelines on Intravenous Fluid Therapy for Adult Surgical Patients (GIFTASUP) recommend appropriate IV fluid prescribing in the pre-operative and postoperative period. Recent prospective surveys, using historical controls, suggested that guidelines and instruction on fluid management could improve outcome [9,18]. Teaching on these important clinical skills should use uniform guidance to avoid inconsistencies, and ideally in clinical surroundings with practical experience, such as “pre-prescribing” [19]. The National Clinical Guideline Centre in the UK has now published guidelines on intravenous fluid therapy [12]. Clearly, such material should be provided for clinical use in several forms (posters, booklets, and in electronic form) and also should be incorporated in relevant undergraduate textbooks.

Knowledge of the basic facts of fluid therapy is not in itself sufficient. Considering his Pyramid model for clinical assessment, Miller argued “it may be reasonable to assume that either action or performance implies achievement of the more basic elements of the triangle” [20]. Although we agree that further instruction is needed to apply this knowledge clinically, we are concerned by the assumption that performance, which may be superficially tested, necessarily provides sufficient evidence of adequate knowledge. The clinical scenarios used in examinations of competence and performance frequently focus on complex problems, and fail to assess the protean tasks routinely required of the newly qualified doctor. We accept that knowledge alone is insufficient to be a practitioner, but it has to form a foundation. Our current assessment methods risk emphasis on process above content. It is of interest that the fluid prescribing tasks used in the examinations in our institution concentrate on prescribing for hypovolaemia and fluid replacement, rather that routine maintenance. This echoes the content of the books we reviewed. We suggest that teaching and assessment include the everyday, and test the whole pyramid from knowledge to performance.

## Conclusion

In – service teaching programmes for trainee doctors have been set up to counter the problem of inadequate knowledge [21]. However, this is illogical and too late: students should graduate with sufficient knowledge to be safe doctors, and to meet this, a range of suitable textbooks is required. Teaching postgraduates has a very small effect, with prescribed sodium intake still greatly in excess of guideline suggestions [22,23]. Currently, most of the books available in the UK are insufficient for these needs, and there is no reason to believe that the UK should be unique in this deficiency. We suggest that more stringent criteria should be applied by authors, publishers, and reviewers when assessing coverage of such important and universal topics.

## Competing interests

The authors declare that they have no competing interests.

## Authors’ contributions

AGMTP developed the research idea and the study plan, collected and scored the books, and wrote the first draft of the text. S P-B assisted with the data presentation and contributed to the writing of the final text. GBD conceived the research idea, assisted with the book collection, reviewed a sample of the books, collated and analysed the data, and wrote the final text. All authors are responsible for the final text. All authors read and approved the final manuscript.

## Pre-publication history

The pre-publication history for this paper can be accessed here:

http://www.biomedcentral.com/1472-6920/14/35/prepub

## Supplementary Material

Additional file 1 Marks given to each book for each question. Click here for file
