# Supplementary material for: Undergraduate medical textbooks do not provide adequate information on intravenous fluid therapy: a systematic survey and suggestions for improvement
Source: BMC Med Educ. 2014 Feb 20;14:35. doi: 10.1186/1472-6920-14-35 (PMC3933276; doi:10.1186/1472-6920-14-35)
Supplement: Additional file 1 — Marks given to each book for each question. [file 1472-6920-14-35-S1.docx]

| book reference symbol: | a | b | c | d | e | f | g | h | i | j | k | l | m | n | o | p | q | r | s | t | u | v | w | x | y | z | 1 | 2 | 3 | **Mean** |
| --- | --- | --- | --- | --- | --- | --- | --- | --- | --- | --- | --- | --- | --- | --- | --- | --- | --- | --- | --- | --- | --- | --- | --- | --- | --- | --- | --- | --- | --- | --- |
| **Normal physiology** |  |  |  |  |  |  |  |  |  |  |  |  |  |  |  |  |  |  |  |  |  |  |  |  |  |  |  |  |  |  |
| Standard requirements | 1 | 2 | 2 | 0 | 0 | 0 | 0 | 0 | 1 | 0 | 1 | 2 | 0 | 2 | 0 | 0 | 0 | 1 | 2 | 2 | 0 | 0 | 2 | 0 | 0 | 0 | 0 | 1 | 2 | 0.72 |
| Fluid balance | 1 | 1 | 2 | 1 | 0 | 1 | 0 | 0 | 2 | 1 | 1 | 1 | 0 | 0 | 0 | 1 | 0 | 1 | 2 | 2 | 0 | 1 | 2 | 1 | 0 | 1 | 0 | 1 | 2 | 0.86 |
| Body fluids | 0 | 1 | 2 | 0 | 0 | 0 | 0 | 0 | 1 | 0 | 0 | 0 | 0 | 0 | 0 | 0 | 0 | 0 | 0 | 0 | 0 | 2 | 0 | 0 | 0 | 2 | 1 | 0 | 0 | 0.31 |
| Routes of loss | 1 | 1 | 2 | 1 | 0 | 0 | 0 | 0 | 1 | 0 | 0 | 1 | 0 | 0 | 0 | 0 | 0 | 0 | 0 | 0 | 0 | 0 | 0 | 0 | 0 | 0 | 0 | 0 | 0 | 0.24 |
| **Effects of surgery** |  |  |  |  |  |  |  |  |  |  |  |  |  |  |  |  |  |  |  |  |  |  |  |  |  |  |  |  |  |  |
| Systemic inflammation | 0 | 0 | 2 | 1 | 0 | 1 | 0 | 0 | 1 | 0 | 0 | 1 | 0 | 2 | 0 | 0 | 0 | 0 | 1 | 1 | 0 | 0 | 0 | 0 | 0 | 0 | 0 | 0 | 0 | 0.34 |
| ADH | 2 | 0 | 2 | 0 | 0 | 1 | 0 | 0 | 2 | 0 | 0 | 1 | 0 | 2 | 0 | 0 | 0 | 1 | 0 | 0 | 0 | 0 | 1 | 0 | 0 | 0 | 0 | 0 | 0 | 0.41 |
| Types of surgery | 0 | 0 | 0 | 0 | 0 | 0 | 0 | 0 | 0 | 0 | 0 | 0 | 0 | 0 | 0 | 0 | 0 | 0 | 0 | 0 | 0 | 0 | 0 | 0 | 0 | 0 | 0 | 0 | 0 | 0 |
| Evaporation | 0 | 0 | 0 | 0 | 0 | 0 | 0 | 0 | 0 | 0 | 0 | 0 | 0 | 0 | 0 | 0 | 0 | 0 | 0 | 0 | 0 | 0 | 0 | 0 | 0 | 0 | 0 | 0 | 0 | 0 |
| Normal postop changes | 1 | 0 | 2 | 0 | 0 | 0 | 0 | 0 | 2 | 0 | 0 | 0 | 0 | 0 | 2 | 2 | 2 | 0 | 0 | 0 | 0 | 0 | 0 | 0 | 0 | 0 | 0 | 0 | 0 | 0.38 |
| **Assessment of losses** |  |  |  |  |  |  |  |  |  |  |  |  |  |  |  |  |  |  |  |  |  |  |  |  |  |  |  |  |  |  |
| Assess blood volume | 1 | 1 | 2 | 1 | 0 | 0 | 1 | 1 | 2 | 1 | 0 | 1 | 0 | 1 | 0 | 0 | 1 | 2 | 0 | 2 | 1 | 1 | 2 | 0 | 0 | 0 | 1 | 1 | 2 | 0.86 |
| Signs of blood loss | 1 | 1 | 2 | 0 | 0 | 0 | 1 | 0 | 1 | 0 | 0 | 1 | 0 | 1 | 1 | 0 | 1 | 0 | 0 | 1 | 0 | 1 | 1 | 0 | 0 | 0 | 1 | 1 | 2 | 0.59 |
| Perioperative loss | 1 | 1 | 2 | 1 | 0 | 0 | 0 | 0 | 1 | 0 | 0 | 0 | 0 | 1 | 0 | 0 | 0 | 0 | 0 | 0 | 0 | 0 | 0 | 0 | 0 | 0 | 0 | 0 | 2 | 0.31 |
| Overload | 1 | 1 | 2 | 1 | 0 | 1 | 0 | 1 | 1 | 1 | 0 | 1 | 0 | 1 | 0 | 0 | 0 | 1 | 0 | 1 | 1 | 1 | 1 | 1 | 0 | 0 | 1 | 1 | 2 | 0.72 |
| Hypovolaemia | 1 | 1 | 2 | 1 | 0 | 1 | 0 | 1 | 2 | 1 | 0 | 1 | 0 | 1 | 0 | 0 | 1 | 1 | 0 | 2 | 1 | 1 | 2 | 1 | 0 | 0 | 1 | 2 | 2 | 0.9 |
| Adequacy of therapy | 1 | 0 | 1 | 0 | 0 | 0 | 0 | 1 | 2 | 0 | 0 | 1 | 0 | 0 | 0 | 0 | 1 | 1 | 0 | 2 | 0 | 2 | 2 | 1 | 0 | 0 | 0 | 2 | 2 | 0.66 |
| **Treatment** |  |  |  |  |  |  |  |  |  |  |  |  |  |  |  |  |  |  |  |  |  |  |  |  |  |  |  |  |  |  |
| Intraoperative | 0 | 0 | 0 | 0 | 0 | 0 | 0 | 0 | 0 | 0 | 0 | 0 | 0 | 0 | 0 | 0 | 0 | 0 | 0 | 0 | 0 | 0 | 0 | 0 | 0 | 0 | 0 | 0 | 0 | 0 |
| Suitable regimes | 1 | 2 | 2 | 1 | 0 | 0 | 0 | 0 | 0 | 0 | 0 | 0 | 0 | 0 | 0 | 0 | 0 | 0 | 0 | 0 | 0 | 0 | 1 | 0 | 0 | 0 | 0 | 0 | 2 | 0.31 |
| Reasons for therapy | 0 | 1 | 2 | 0 | 0 | 0 | 0 | 1 | 1 | 0 | 0 | 0 | 0 | 1 | 0 | 1 | 0 | 1 | 0 | 1 | 0 | 2 | 1 | 0 | 0 | 0 | 0 | 0 | 0 | 0.41 |
| Resuscitate GI losses | 1 | 1 | 2 | 1 | 0 | 1 | 1 | 0 | 1 | 0 | 0 | 1 | 0 | 2 | 0 | 0 | 0 | 0 | 0 | 1 | 0 | 1 | 1 | 0 | 0 | 0 | 1 | 2 | 2 | 0.66 |
| Resuscitate Blood loss | 1 | 2 | 2 | 1 | 0 | 1 | 1 | 1 | 1 | 1 | 0 | 1 | 1 | 1 | 1 | 1 | 1 | 1 | 0 | 1 | 0 | 1 | 1 | 0 | 0 | 0 | 1 | 2 | 2 | 0.9 |
| Resuscitate sepsis | 1 | 2 | 2 | 1 | 0 | 1 | 2 | 1 | 1 | 0 | 0 | 1 | 0 | 1 | 0 | 0 | 0 | 1 | 0 | 1 | 0 | 2 | 2 | 0 | 0 | 0 | 1 | 2 | 2 | 0.83 |
| **Fluid preparations** |  |  |  |  |  |  |  |  |  |  |  |  |  |  |  |  |  |  |  |  |  |  |  |  |  |  |  |  |  |  |
| 5 % Dextrose | 0 | 2 | 2 | 2 | 0 | 2 | 0 | 0 | 0 | 0 | 0 | 0 | 0 | 0 | 0 | 2 | 0 | 0 | 0 | 2 | 0 | 1 | 2 | 0 | 0 | 0 | 0 | 0 | 1 | 0.55 |
| 0.9 % w/v saline | 0 | 2 | 2 | 2 | 0 | 2 | 0 | 0 | 0 | 0 | 0 | 0 | 0 | 2 | 0 | 2 | 0 | 0 | 0 | 2 | 0 | 2 | 2 | 0 | 0 | 0 | 0 | 0 | 1 | 0.66 |
| Hartmanns/ Ringer lactate | 0 | 1 | 2 | 2 | 0 | 1 | 0 | 0 | 0 | 0 | 0 | 0 | 0 | 2 | 0 | 2 | 0 | 0 | 0 | 2 | 0 | 2 | 2 | 0 | 0 | 0 | 0 | 0 | 1 | 0.59 |
| Gelofusin | 0 | 2 | 2 | 0 | 0 | 1 | 0 | 0 | 0 | 0 | 0 | 0 | 0 | 0 | 0 | 1 | 0 | 0 | 0 | 0 | 0 | 1 | 2 | 0 | 0 | 0 | 0 | 0 | 1 | 0.34 |
| Human Albumin solution | 0 | 0 | 2 | 0 | 0 | 0 | 0 | 0 | 0 | 0 | 0 | 0 | 0 | 0 | 0 | 2 | 0 | 0 | 0 | 0 | 0 | 1 | 2 | 0 | 0 | 0 | 0 | 0 | 1 | 0.28 |
| **Sum score** | 16 | 25 | 45 | 17 | 0 | 14 | 6 | 7 | 23 | 5 | 2 | 14 | 1 | 20 | 4 | 14 | 7 | 11 | 5 | 23 | 3 | 22 | 29 | 4 | 0 | 3 | 8 | 15 | 29 |  |

Marks given to each book for each question
